# Direct Visualization of Metal-Induced Gap State Distribution and Valley Band Evolution at Metal Versus Semimetal MoS_2_ Interfaces

**DOI:** 10.1021/acsnano.5c03676

**Published:** 2025-05-15

**Authors:** Yi-Feng Chen, Hung-Chang Hsu, Hao-Yu Chen, Liang-Yu Chen, Yan-Ruei Lin, Ming-Yang Li, Iuliana P. Radu, Ya-Ping Chiu

**Affiliations:** † Graduate School of Advanced Technology, 33561 National Taiwan University, Taipei 10617, Taiwan; ‡ Department of Physics, National Taiwan University, Taipei 10617, Taiwan; § 63393 Taiwan Semiconductor Manufacturing Company, Hsinchu 30078, Taiwan; ∥ Institute of Physics, Academia Sinica, Taipei 115201, Taiwan; ⊥ Institute of Atomic and Molecular Sciences, Academia Sinica, Taipei 10617, Taiwan

**Keywords:** interlayer coupling, metal-induced gap state, valley band modulation, contact engineering, scanning tunneling microscopy

## Abstract

The interlayer coupling between metals and the two-dimensional (2D) semiconductors’ conduction band (CB), encompassing metal-induced gap states (MIGS) and valley band modulation, critically influences both the Schottky barrier height (SBH) and intrinsic sheet resistance. Understanding the CB modulation induced by metals/semimetals is, therefore, essential for contact engineering optimization. Given that the MIGS decay length and orbital interactions are spatially confined to the nanoscale region proximate to the 2D semiconductor interface, we employed scanning tunneling microscopy/spectroscopy to quantitatively determine the MIGS decay length and CB minimum on various metal/semimetal substrates. This approach enabled the comprehensive characterization of MIGS distribution, charge neutrality level variation, and SBH properties. Our findings demonstrate that maintaining valley band structure integrity during semimetal interlayer coupling facilitates reduced intrinsic sheet resistance. These results elucidate the mechanism underlying weak interlayer coupling at semimetal–2D semiconductor junctions and their superior contact transport performance, providing insights into the rational design of future 2D-based devices.

## Graphical abstract

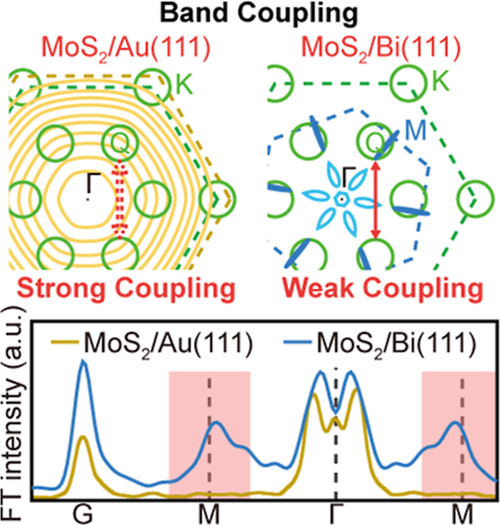

## Introduction

Recent extensive investigations into the electronic properties of two-dimensional (2D) semiconductors have revealed persistent unexplained variations and unresolved challenges in 2D heterojunction systems, primarily attributed to complex interlayer coupling mechanisms.^1–6^ In metal–semiconductor junctions (MSJ), metal-induced band modulation near the conduction band (CB) of 2D semiconductors, particularly transition metal dichalcogenides (TMDs), significantly influences contact transport performance.^7–9^ Two critical phenomena emerge: below the CB, metal-induced gap states (MIGS) lead to Fermi level pinning and substantial Schottky barrier height (SBH);^10,11^ above the CB, the TMDs’ **Q**-valley band exhibits greater susceptibility to contacted metals compared to **K**-valleys, affecting intrinsic sheet resistance and enabling superconductivity through electron–phonon and spin–orbital coupling.^12–14^ Consequently, MIGS and **Q**-valley band characteristics serve as crucial indicators for identifying optimal metal candidates in contact engineering for 2D-based devices.

Phase engineering,^15^ van der Waals contacts with 2D materials,^16–20^ ultrahigh vacuum metal deposition,^21^ and doping strategies have been shown to effectively modulate interlayer coupling and reduce contact resistance.^22,23^ A recent seminal study demonstrated that bismuth (Bi) electrodes in MoS_2_-based devices achieve reduced contact resistance (123 Ω μm) due to their distinctive MIGS energy distribution.^9^ The unique MIGS configuration in MoS_2_/Bi systems is hypothesized to concentrate away from MoS_2_’s conduction band minimum (CBM), achieving gap-state saturation.^9^ This absence of MIGS near the CBM stems from weak interlayer coupling with Bi substrates and subsequent valley band modulation on its CB.^13,24^ The minimal valley band modulation in MoS_2_/Bi systems better preserves the **Q**-valley band compared to metal substrates, resulting in lower intrinsic sheet resistance.^9,12,25^ Thus, understanding band modulation under interlayer coupling between metals/semimetals and 2D semiconductors’ CB is fundamental for advancing 2D-based device development. However, due to the MIGS decay length and orbital interaction constraints, both MIGS and valley band modulation are confined to nanoscale regions near junction interfaces, necessitating subnanoresolved microscopy with electronic spectroscopy capabilities for comprehensive characterization.

In this investigation, we employed scanning tunneling microscopy/spectroscopy (STM/S) to achieve the atomic-scale visualization of MIGS decay properties in MoS_2_/Au­(111) and MoS_2_/Bi­(111) systems. The Au(111) and Bi(111) substrates were selected as prototypical metal and semimetal interfaces, respectively, characterized by contrasting properties of MIGS density (high/low) and interlayer coupling strength (strong/weak). Our findings demonstrated that the MIGS distribution in MoS_2_/Bi­(111) was substantially displaced from its CBM, indicating gap-state saturation and weak interlayer coupling for its valley band.^9^ Additionally, we directly observed valley band destruction in MoS_2_/Au­(111) through quasiparticle interference (QPI), confirming a relatively strong interlayer coupling. Conversely, MoS_2_/Bi­(111) exhibited a preserved valley band structure and elevated carrier concentration, supporting reduced intrinsic sheet resistance. This study provides direct comparative evidence of MIGS distribution on metal/semimetal substrates and elucidates the valley band mechanisms underlying superior contact performance in semimetal-2D semiconductor junctions, offering insights for future 2D-based device design optimization.

## Results and Discussion

In this work, we performed STM/S measurements on monolayer MoS_2_ with Au(111) and Bi(111) substrates to systematically compare substrate-induced interlayer coupling effects. The (111) lattice orientation of metals/semimetals, with its 6-fold symmetry, matches that of MoS_2_, which can further reduce in-plane contact performance anisotropy and enhance the stability of contact performance.^3,12^ Moreover, the well-ordered atomic arrangements of these substrate surfaces minimize electronic variations attributed to surface roughness.^26^ Figure 1a,b presents large-scale STM images revealing distinctly different morphologies between MoS_2_/Au­(111) and MoS_2_/Bi­(111) systems. While both surfaces exhibit terraces, in general, the surface morphology of MoS_2_/Au­(111) reveals numerous pits with diameters of approximately 3 nm, whereas MoS_2_/Bi­(111) demonstrates a comparatively uniform morphology. Previous studies have identified the numerous small pit regions as substrate-originated pits of MoS_2_/Au­(111).^27^ Additionally, the height profile analysis in the inset of Figure 1a reveals a step height difference (*d*_Au_) of 2.4 ± 0.1 Å along the blue arrow, consistent with Au(111) step height.^28,29^ Similarly, the inset of Figure 1b shows atomically flat morphologies with a step height (*d*_Bi_) of approximately 4.0 ± 0.1 Å, characteristic of Bi(111) terraces.^30^ These observations strongly suggest that the overall morphology is predominantly determined by the underlying substrate characteristics.

**Figure 1:**
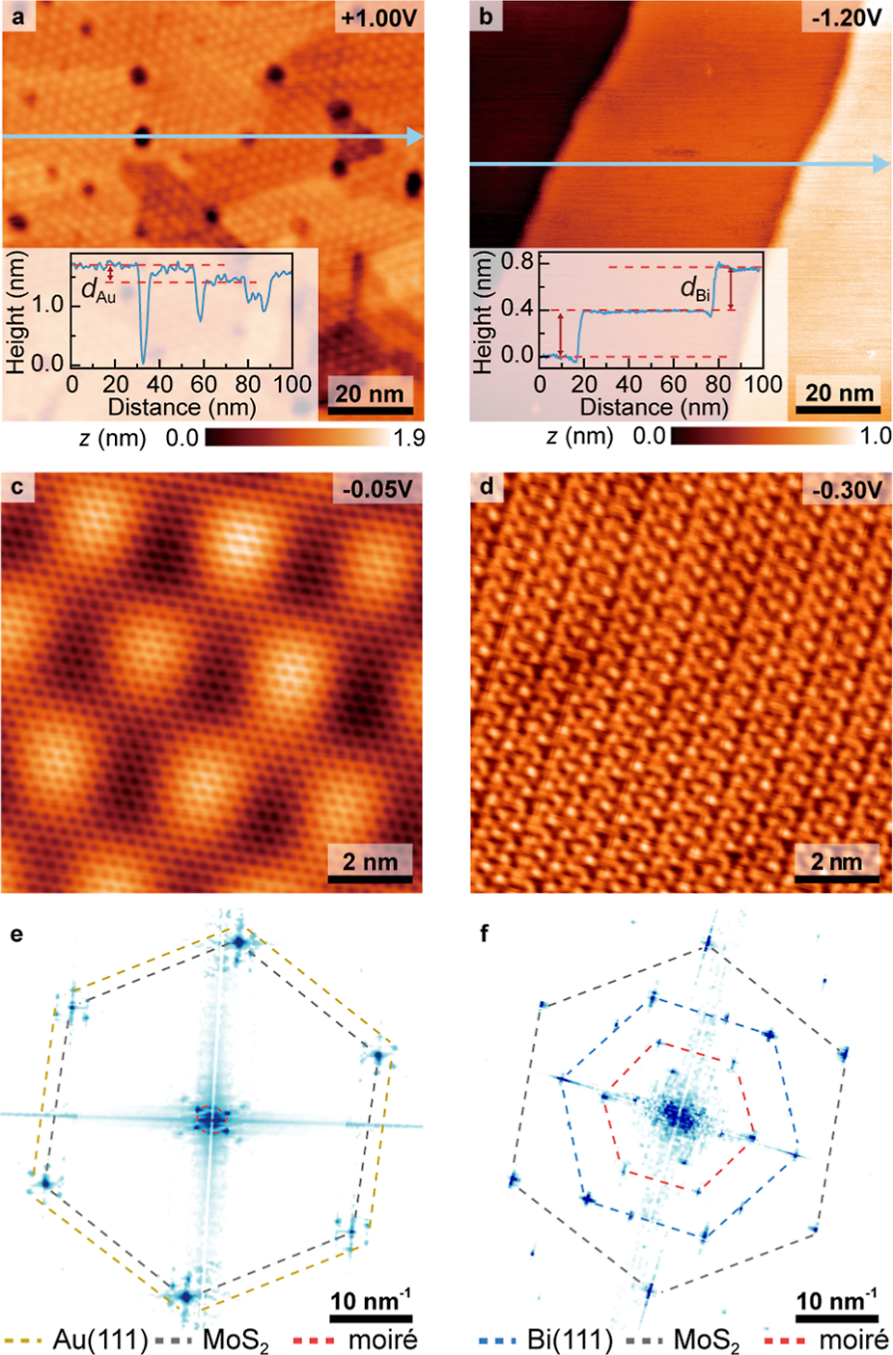
(a) Large-scale STM image of MoS_2_/Au­(111) is measured with *V*_s_ = +1.00 V, *I*_set_ = 500 pA; the inset shows the height profile along the blue arrow. The Au(111) step height is denoted as *d*_Au_ = 2.4 ± 0.1 Å. (b) Large-scale STM image of MoS_2_/Bi­(111) is measured with *V*_s_ = −1.20 V, *I*_set_ = 200 pA, and the inset shows the height profile along the blue arrow. The Bi(111) step height is denoted as *d*_Bi_ = 4.0 ± 0.1 Å. (c) Atomic-scale STM image of MoS_2_/Au­(111) is measured with *V*_s_ = −0.05 V, *I*_set_ = 950 pA. (d) Atomic-scale STM image of MoS_2_/Bi­(111) is measured with *V*_s_ = −0.30 V, *I*_set_ = 100 pA. (e,f) FT patterns correspond to (c,d), respectively.

High-resolution imaging of the atomic arrangements for MoS_2_/Au­(111) and MoS_2_/Bi­(111) is presented in Figure 1c,d, with their corresponding Fourier transformation (FT) patterns shown in Figure 1e,f, respectively. The FT patterns reveal corresponding hexagonal signals for both Au and Bi substrates (marked as yellow/blue dashed hexagons in Figure 1e,f, respectively), supporting the idea that our metallic/semimetallic substrates are aligned along the (111) lattice orientation. Furthermore, an analysis of the FT patterns reveals that MoS_2_ and Au(111) exhibit nearly aligned stacking (Figure 1e), while MoS_2_/Bi­(111) demonstrates a rotational misalignment of approximately 20° (Figure 1f). The observed reciprocal lattice vector of moiré patterns ***G*_M_**, from the center to the corner of the red dashed hexagon in Figure 1e,f, align well with the theoretical formula ***G***_**M**_ = ***G***_TMD_ – ***G***_substrate_.^2,31,32^ These results demonstrate that both the macroscopic morphology and nanoscale moiré patterns are fundamentally influenced by substrate–overlayer interactions.

To elucidate how our metallic/semimetallic substrates modify the electronic properties of MoS_2_, we investigated the characteristics of MIGS arising from the electronic coupling between MoS_2_ and the substrate. Previous research has established that MIGS-dominated energy levels exhibit finite decay lengths (10^–1^ to 10^0^ nm),^33–35^ in contrast to the significantly longer decay lengths of normal Bloch states.^34,36^ In the substrate-contacted region, the uniform characteristics of MIGS make it difficult to quantitatively determine their decay behavior. At fixed energy levels, MIGS exhibit characteristic exponential decay (*e* ^–***q*** *x*^, where ***q*** is the decay constant) as MIGS propagates into the substrate-free region (noncontacted region).^34^ To overcome this limitation and characterize MIGS effectively, we analyzed regions near the substrate edge where the transition occurs from complete contact to suspension as MoS_2_ crosses the substrate terrace. The aforementioned substrate-originated pits provide a suitable structure for the substrate-contacted/-suspended regions, making them ideal for investigating the MIGS decay behavior.

As illustrated in Figure 2a, a schematic diagram depicts an atomic model of a single-layer MoS_2_ film both in contact with and suspended from the Au(111) substrate. To examine the substrate–film interlayer coupling and its induced electronic effects on the MoS_2_ film, we designated the contact point between the film and substrate edge as the origin, “0”, in Figure 2a. This origin position, where the substrate-contacted and substrate-free regions meet, serves as a reference point for studying how substrate interactions spatially modulate the electrical properties of the monolayer MoS_2_ film.

**Figure 2:**
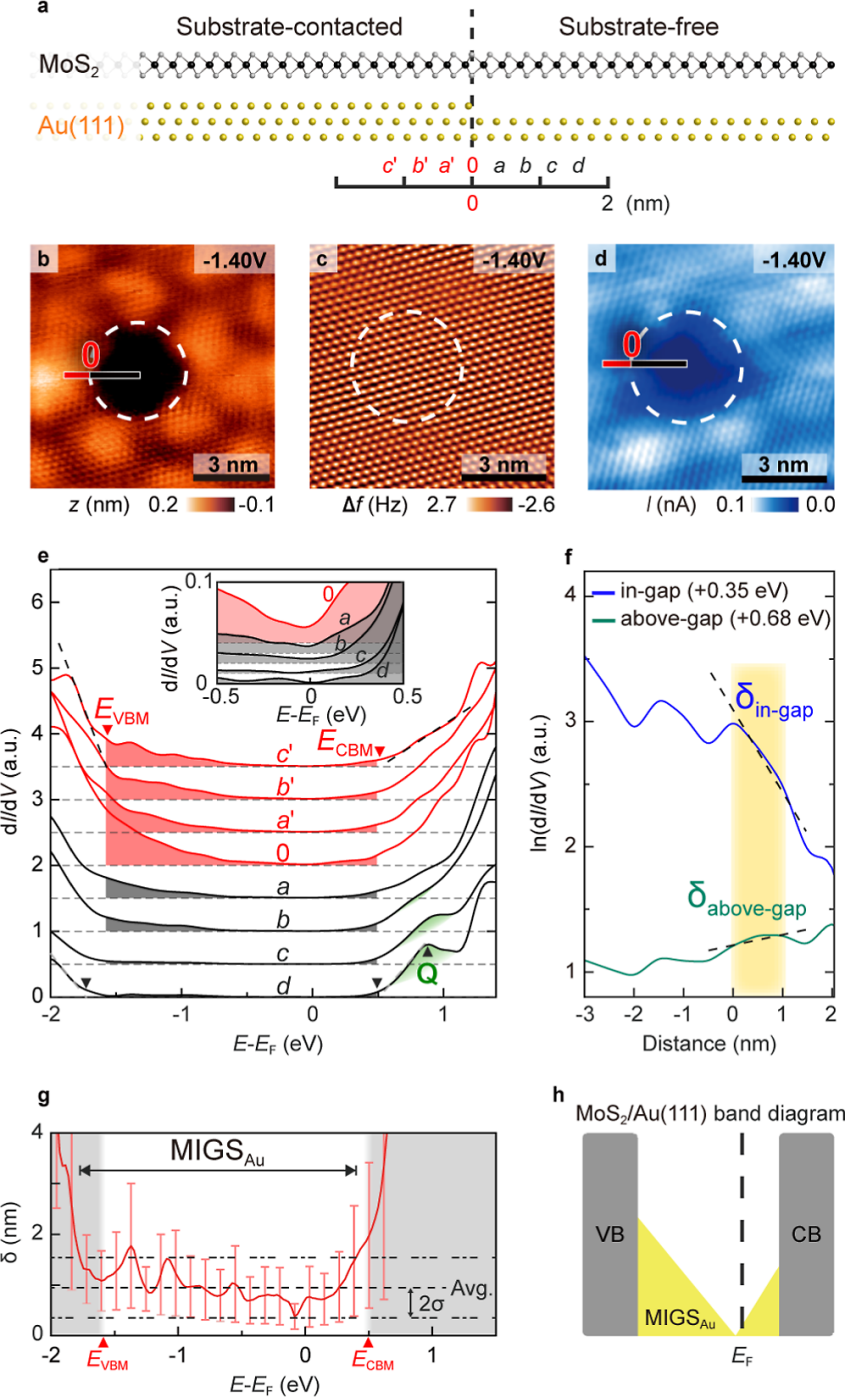
(a) Side view atomic schematic shows the substrate-contacted and substrate-free region in MoS_2_/Au­(111). The substrate edge is regarded as the origin “0”. (b) Constant current mode of STM image is measured adjacent to the pit (substrate-free) region (marked by the white dashed line) with *V*_s_ = −1.40 V, *I*_set_ = 310 pA. (c,d) Constant height nc-AFM and in situ STM current (*I*) images are measured adjacent to the Au(111) substrate edge (white dashed line) of MoS_2_/Au­(111) with *V*_s_ = −1.40 V, and the Au(111) substrate edge is regarded as the origin. (e) d*I*/d*V* curves derived from the substrate-contacted (“*a*′” to “*c*′” curves) to substrate-free region (“*a*” to “*d*” curves) are along the red and black bar in (b,d) with a spatial resolution of approximately 0.4 nm, respectively. The inset shows the enlarged d*I*/d*V* curves from the origin (“0” curve) to the substrate-free region (“*a*” to “*d*” curves) at the energy level near the *E*_F_. The CBM and VBM of curves “*c*′” and “*d*” derived from linear fitting are marked by inverted triangles with corresponding colors. The linear fitting is shown as the dashed line. The “**Q**” peak marked by the black triangle appears at the “*d*” curve with an energy level of +0.80 eV. (f) Logarithm of d*I*/d*V* curves are taken at the energy level in the band gap (+0.35 eV, blue line) and above the band gap (+0.68 eV, green line). The yellow part represents the linear decay region (0 to 1 nm). The black dashed lines are linear fitting results to derive the decay length δ. (g) Plots of decay length δ correspond to different energy levels. The MIGS energy distribution (the black arrow) is defined as the energy level range, where the decay length approaches the average decay length within two standard deviation ranges (marked by the black dashed line). The gray parts represent the MoS_2_ CB and VB in the substrate-contacted region. (h) Schematic viewgraph shows the band diagram and MIGS energy distribution of substrate-contacted region in MoS_2_/Au­(111).

Figure 2b presents an STM image of a single-layer MoS_2_ film on an Au(111) surface adjacent to a pit. To verify the presence of monolayer MoS_2_ over the Au(111) surface pit and examine how substrate contact affects its electronic structure, Figure 2c,d displays noncontact atomic force microscopy (nc-AFM) and the STM current (*I*) images of the MoS_2_/Au­(111) region near the pit. These measurements are obtained in constant height mode with a fixed sample bias (*V*_s_ = −1.40 V).

The Au(111) surface pit induces changes in the apparent height, as reflected in the STM image (Figure 2b). This structural variation corresponds to a significant reduction in current in the pit region, as observed in the STM current image (Figure 2d). However, simultaneous nc-AFM measurements (Figure 2c) reveal a continuous MoS_2_ structure across the pit area. This observation indicates that the current reduction in the STM current mapping primarily originates from morphological changes in the underlying substrate rather than in the MoS_2_ layer. As illustrated in the atomic model in Figure 2a, this region enables experimental investigation of how substrate contact modulates the electronic structure of monolayer MoS_2_.^27^

In Figure 2b, the white dashed line marks the substrate edge as the origin. The d*I/*d*V* spectral data were collected with a spatial resolution of approximately 0.4 nm from regions where MoS_2_ contacts the substrate (red bar) and the pit area without substrate contact (black bar). These measurements are correspondingly displayed as red and black curves in Figure 2e. As shown in Figure 2e, curves “*a*′” through “*c*′” represent d*I*/d*V* spectra obtained from the substrate-contacted region, while curves “0” through “*d*” depict d*I*/d*V* measurements acquired along the black bar extending from the origin into the pit area. The inset in Figure 2e highlights curves “0” through “*d*” from −0.5 eV to +0.5 eV.

According to the linear regression analysis method in Supporting Information 1, it reveals that, in the substrate-free region, the CBM is +0.50 ± 0.02 eV, and the valence band maximum (VBM) is −1.72 ± 0.01 eV.^37^ In the substrate-contacted region, the CBM and VBM values are +0.51 ± 0.05 eV and −1.58 ± 0.05 eV, respectively.^12^ In the d*I*/d*V* spectral data of the MoS_2_-substrate contact area (Figure 2e), the curves “*a*′” through “*c*′” show consistent behavior on intensive in-gap states (direct comparison is shown in Supporting Information 2). These in-gap states are also the main reason for the nonzero current in Figure 2d using the in-gap sample bias (*V*_s_ = −1.40 V). In regions where MoS_2_ lacks substrate contact, the d*I/*d*V* curves (“*a*” through “*d*” in Figure 2e and the inset) exhibit progressively decreasing in-gap states highlighted by colored shading between the overlapping band gap (−1.58 eV to +0.50 eV) and the corresponding *x*-axis (direct comparison is shown in Supporting Information 2). The gradually reduced d*I*/d*V* intensity along the curve from “*a*” to “*d*” corresponds to decreased in-gap states, suggesting the characteristic of MIGS. MoS_2_ in the substrate-free region characteristically should exhibit a peak “**Q**” representing **Q**-valley at approximately +0.80 eV.^38,39^ This distinctive feature only emerges in spectrum “*d*” with a distance between 1.2 and 1.6 nm (3 to 4 × 0.4 nm) after the in-gap states decay. The presence of MIGS proposes to exhibit exponential decay behavior within a specific spatial distribution range. The spatial range of the MIGS decay behavior is typically quantified using the decay length δ = 1/***q***.^36^

To quantitatively investigate the spatial distribution of MIGS, Figure 2f presents the logarithmic d*I*/d*V* (ln­(d*I*/d*V*)) curves, revealing two distinct behaviors at constant energy levels from substrate-contacted to substrate-free MoS_2_. Using the substrate edge as a reference point, the energy level above the band gap (green line, +0.68 eV) shows minimal variation near the origin and represents the significantly large decay length δ _**above‑gap**_. However, within the band gap (blue line, +0.35 eV), the curve exhibits linear decay characteristics within approximately 1 nm from the origin to substrate-free MoS_2_ and indicates a decay length δ _**in‑gap**_ of around 1.5 nm.

Compilation of decay lengths across different energy intervals in Figure 2g demonstrates that decay lengths diverge near the VBM and CBM, indicating the Bloch state dominance. Within the band gap, however, decay lengths oscillate between 0.5 and 1.5 nm, consistent with the MIGS decay length of MoS_2_.^36^

To explain the SBH mechanism, we identified the MIGS distribution through statistical analysis.^24,36^ The dashed line in Figure 2g represents the average decay length near the minimum value, with boundaries set at two standard deviations from the mean. Using this decay length criterion, the observed MIGS distribution marked as the black arrow (−1.74 eV to +0.41 eV) spans nearly the entire MoS_2_ band gap in the substrate-contacted region, extending from the CB and valence band (VB) (Figure 2h).^36^ The MIGS distribution extending from the CB and VB makes the charge neutrality level (CNL) fixed in the band gap, resulting in a significant SBH.^24,40^

Next, the SBH, defined as the energy difference between the semiconductor’s Fermi level (*E*_F_) and CBM in the substrate-contacted region, was determined to be +0.51 eV for the Au(111)-MoS_2_ interface. This value, derived from the CBM of the metal-contacted MoS_2_ region, supports the gap state pinning mechanism.^9,41,42^ Furthermore, in the substrate-contacted region where MIGS occurs, the characteristic **Q**-valley peak at approximately +0.80 eV above the CBM vanishes, indicating strong CB interlayer coupling. These observations demonstrate that the poor contact resistance characteristics arise from the combination of strong MoS_2_–Au­(111) interlayer coupling, extensive MIGS distribution within the band gap, and a large SBH.

Similar experiments on MoS_2_/Bi­(111) revealed unique electronic properties when MoS_2_ contacts a semimetallic substrate. Figure 3a illustrates the atomic model geometry of monolayer MoS_2_ with and without contact with a semimetallic Bi(111) substrate, with the origin defined at the Bi substrate edge.

**Figure 3:**
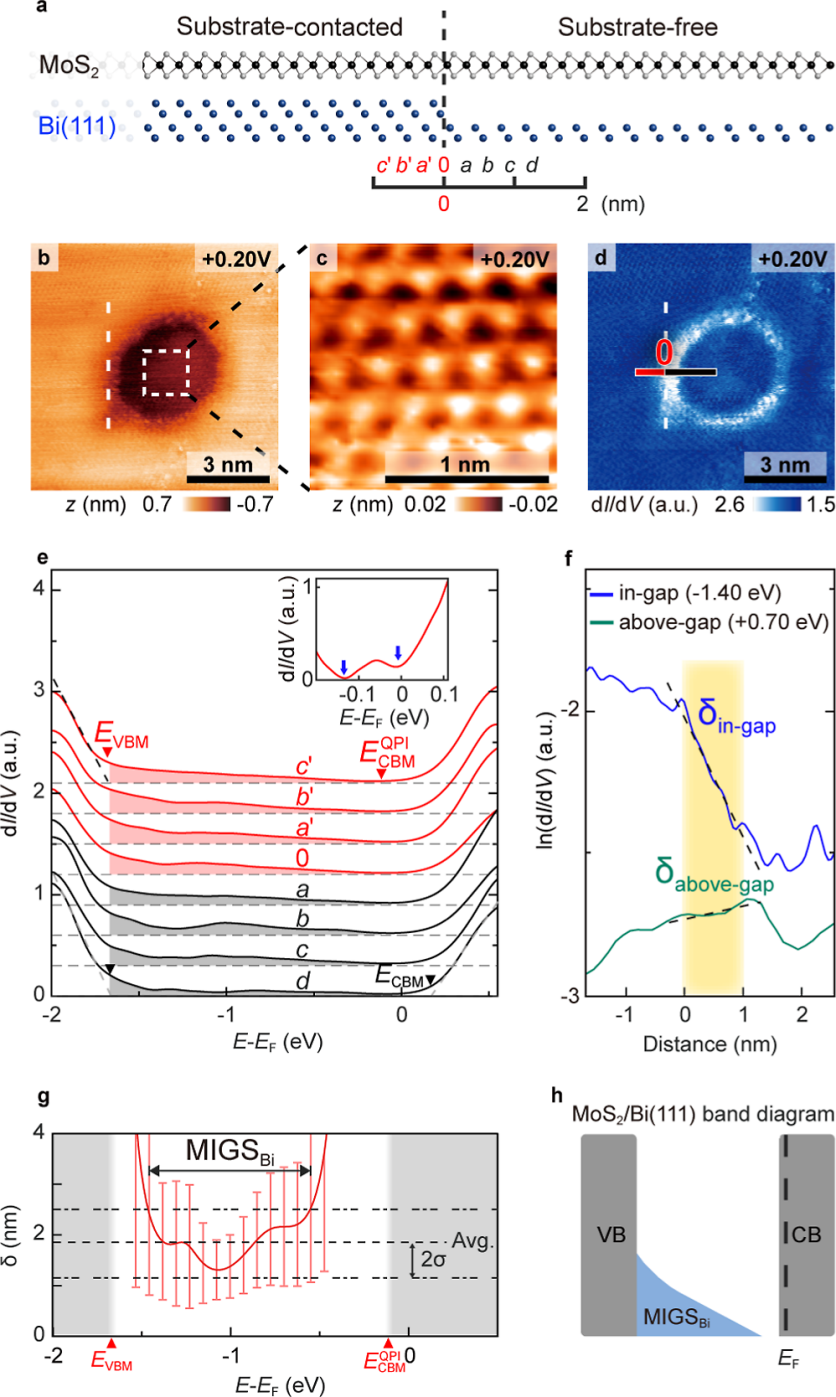
(a) Side view atomic schematic shows the substrate-contacted and substrate-free region in MoS_2_/Bi­(111). (b,d) Constant current STM image and in situ d*I*/d*V* images are measured adjacent to the pit (substrate-free) region of MoS_2_/Bi­(111) with *V*_s_ = +0.20 V *I*_set_ = 300 pA, and the pit’s edge at the left side is marked as the white dashed line and the origin. (c) Enlarged STM image is derived from the white dashed square in (b) and represents the clear MoS_2_ lattice. (e) d*I*/d*V* curves derived from the substrate-contacted (“*a*′” to “*c*′” curves) to substrate-free region (“*a*” to “*d*” curves) are along the red and black bar in (d) with a spatial resolution of approximately 0.3 nm, respectively. The inset shows a detailed d*I*/d*V* curve near the *E*_F_ on the substrate-contacted region of MoS_2_/Bi­(111), and the local minimum is marked by the blue arrow near the *E*_F_ and −0.11 eV. Except for the CBM of curve “*c*′”, the CBM and VBM of curves “*c*′” and “*d*” derived from linear fitting are marked by inverted triangles with corresponding colors. The linear fitting is shown as the dashed line. (f) Logarithm of d*I*/d*V* curves are taken at the energy level in the band gap (−1.40 eV, blue line) and above the band gap (+0.70 eV, green line). The yellow part represents the linear decay region (0 to 1 nm). The black dashed lines are linear fitting results to derive the decay length δ. (g) Plots of decay length δ correspond to different energy levels. The gray parts represent the MoS_2_ CB and VB in the substrate-contacted region. (h) Schematic viewgraph shows the band diagram and MIGS energy distribution of substrate-contacted region in MoS_2_/Bi­(111).

Figure 3b presents the STM topography of monolayer MoS_2_ with and without the semimetallic Bi(111) substrate contact. Figure 3c shows an enlarged STM image from Figure 3b to clearly identify the atomic structure of substrate-free MoS_2_, while Figure 3d presents the d*I*/d*V* mapping corresponding to that in Figure 3b. In the substrate-free region, both the atomic-resolution STM image (Figure 3c) and d*I*/d*V* mapping (Figure 3d) demonstrate continuous MoS_2_ lattice periodicity, indicating uninterrupted MoS_2_ coverage across the Bi(111) pit. This enables investigation of how semimetallic substrate contact affects MoS_2_’s electronic properties.

Using the substrate edge as the origin (Figure 3d), d*I*/d*V* spectra were collected from substrate-contacted (red bars) and substrate-free regions (black bars), shown as red and black curves in Figure 3e. Curves “*a*′” and “*c*′” represent substrate-contacted regions, while curves “0” to “*d*” span from the origin into the substrate-free region. The d*I/*d*V* evolution was measured at 0.3 nm intervals, as shown in Figure 3e.

In the substrate-contacted region, detailed d*I*/d*V* measurements near the *E*_F_, as shown in the inset of Figure 3e, reveal the local minimum near the *E*_F_ and −0.13 eV marked by the blue arrow. Proposing the CBM position obtained through the linear regression analysis,^37^ the analysis shows the CBM values for substrate-contacted and substrate-free regions are +0.15 ± 0.01 eV and +0.16 ± 0.01 eV, respectively, with corresponding VBM values of −1.68 ± 0.01 eV and −1.66 ± 0.01 eV. If the CBM value is adopted, the CBM of substrate-contacted MoS_2_ indicates a SBH of +0.15 eV for MoS_2_/Bi­(111), contradicting previous reports of ohmic contact behavior.^9^ This discrepancy may arise from the suppressed local density of states (LDOS) near *E*_F_ (the local minimum in the inset of Figure 3e), attributed to increased carrier concentration complicating accurate CBM determination.^43,44^

Previous studies of ohmic contact and degenerate semiconductor behavior suggest that the CBM of MoS_2_/Bi­(111) is below the *E*_F_, indicating a high carrier concentration.^9^ Under high carrier concentration, the LDOS near the *E*_F_ could be suppressed, leading to a nonmonotonic increase in the LDOS of the CB and overestimating the actual CBM in linear regression analysis (detailed discussion is in Supporting Information 3). Therefore, QPI, a precise valley band position analysis, is required to validate the aforementioned hypothesis and confirm its consistency with the observed ohmic contact behavior.^45,46^ Supporting Information 3 reveals a persistent 2 × 2 signal at the **M** point in the first Brillouin zone (BZ) above −0.11 eV, attributed to **Q**-valley scattering.^45^ Previous studies indicate that, at carrier concentrations exceeding 10^13^ cm^–2^, the **Q**-valley approaches below *E*_F_ and converges toward the **K**-valley.^9,47^ These observations suggest near-alignment of **Q**- and **K**-valleys, with the CBM of substrate-contacted MoS_2_ at approximately −0.11 eV, characteristic of a degenerate semiconductor behavior. Furthermore, analysis of d*I/*d*V* spectra in Figure 3e from positions “*a*” to “*d*” reveals decay of the in-gap states between −1.66 eV and −0.11 eV (highlighted by colored red/gray shading between the CBM/VBM and *x*-axis) as distance increases from the semimetallic Bi substrate edge into the substrate-free region, indicating the presence of residual MIGS.

To quantify the presence of residual MIGS, ln­(d*I*/d*V*) analysis was performed. ln­(d*I*/d*V*) analysis (Figure 3f) examining decay length δ behavior demonstrates: (1) ln­(d*I*/d*V*) of the minimal variation and extremely large decay length δ _**above‑gap**_ outside the band gap (green curve, +0.70 eV), and (2) characteristic linear decay within 1 nm from the semimetallic substrate edge within the band gap (blue curve, −1.40 eV), similar to MoS_2_/Au­(111) behavior.

The decay length analysis across different energies (Figure 3g) reveals distinct features: (1) MIGS distribution is confined to −1.46 eV to −0.57 eV, much narrower than MoS_2_/Au­(111) (−1.74 eV to +0.41 eV); (2) mean decay length and standard deviations (1.1 to 2.5 nm) exceed MoS_2_/Au­(111) values, suggesting reduced MIGS density in MoS_2_/Bi­(111) (detailed discussion about MIGS density is in Supporting Information 4); (3) MoS_2_/Bi­(111) MIGS concentrate near the VBM rather than CBM, consistent with previous predictions,^9^ attributed to weak interlayer coupling with Bi(111) bands leading to MIGS saturation;^9^ (4) gap state saturation shifts the CNL toward CBM, potentially yielding zero SBH—deviating from previously typical Fermi-level pinning assumption, as shown in Figure 3h.^36,48,49^ MoS_2_/Au­(111) exhibits strong coupling at both the CB and VB, while MoS_2_/Bi­(111) shows weaker coupling, particularly at the CB, corroborated by **Q**-valley peak suppression in MoS_2_/Au­(111) (Figure 2e) versus preserved behavior in MoS_2_/Bi­(111) (Supporting Information 3).

To gain a deeper understanding of the physical mechanism of the weak coupling between MoS_2_ and Bi(111), Figure 4a presents the d*I/*d*V* image of MoS_2_/Au­(111) and defect-induced QPI patterns at *V*_s_ = +0.80 V (corresponding to the “**Q**” peak energy in Figure 2e).^50^ Similar measurements for MoS_2_/Bi­(111) at *V*_s_ = +0.20 V are shown in Figure 4b. The associated FT analysis (Figure 4c,d, respectively) reveals periodic signals related to QPI patterns.^45,46^ Figure 4e,f shows the constant energy contour (CEC) analysis of MoS_2_/Au­(111) and MoS_2_/Bi­(111) corresponding to the energy level of Figure 4a,b.^12,51^

**Figure 4:**
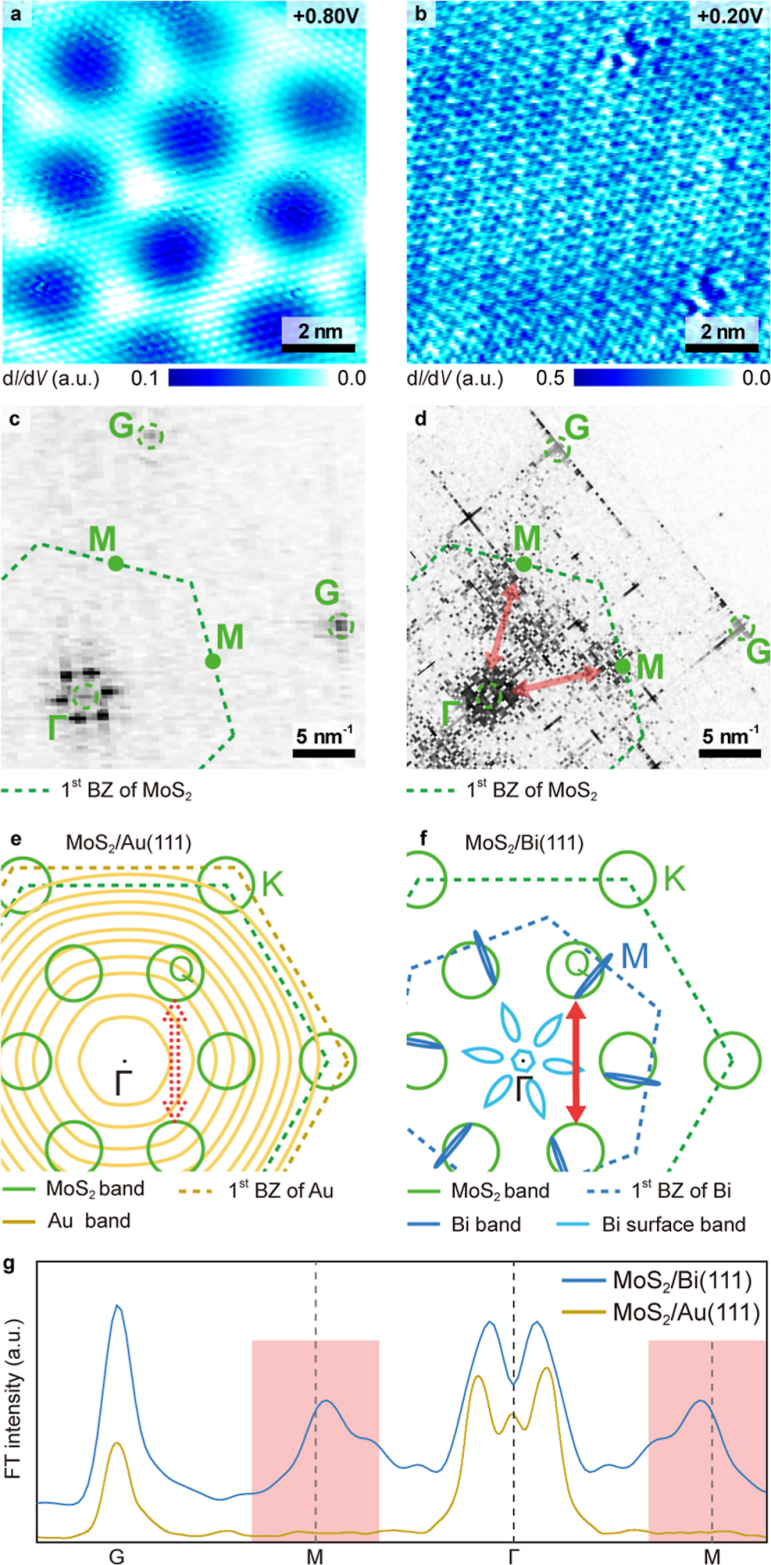
(a) d*I*/d*V* mapping is measured with *V*_s_ = +0.80 V on MoS_2_/Au­(111). (b) d*I*/d*V* mapping is measured with *V*_s_ = +0.20 V on MoS_2_/Bi­(111). (c) and (d) FT images corresponding to (a) and (b), respectively. The green dashed line represents the first BZ of MoS_2_, and the red arrow in (d) represents the 2 × 2 QPI signals. (e) and (f) CEC schematic viewgraph shows the energy band in reciprocal space with the energy level corresponding to (a) and (b), respectively. The red arrow in (f) represents the intervalley scattering of **Q**-valleys, corresponding to the 2 × 2 QPI signals in (d). (g) FT intensity profile is taken along the direction from **Γ** to **G** points in (c) and (d).

CEC analysis of MoS_2_/Au­(111) above CBM (Figure 4e) revealed extensive band overlap between Au(111) and **Q**-valley within MoS_2_’s first BZ, confirmed by the absence of **Q**-valley characteristic peaks in Figure 2e.^12^ The FT intensity profile along **Γ** to **G** (yellow line, Figure 4g) lacks characteristic peaks at **M** points typically seen in 2 × 2 QPI patterns, indicating suppressed **Q**-valley mediated scattering of MoS_2_/Au­(111) (dashed red arrow, Figure 4e). Local FT analysis of pristine and pit regions (Supporting Information 5) confirms persistent 2 × 2 signals in pit areas of MoS_2_/Au­(111), with energy alignment to recovered **Q**-valley characteristic peaks.

In contrast, Figure 4f demonstrates that when MoS_2_ contacts the semimetallic Bi substrate, the band overlap between Bi(111) and MoS_2_ within the first BZ is significantly reduced compared to Au(111), even considering surface states.^51^ This reduced overlap primarily results from the characteristically low LDOS near *E*_F_ in semimetallic Bi(111). Consequently, the **Q**-valley band structure remains better preserved, maintaining intervalley scattering processes marked by the red arrow in Figure 4d,f. This preservation is evidenced by the pronounced 2 × 2 QPI pattern at the **M** point in the MoS_2_/Bi­(111) FT intensity profile (blue curve, Figure 4g). Supporting Information 3 shows this 2 × 2 pattern vanishing below −0.11 eV, consistent with theoretical predictions of **Q**-valley scattering.^9,14^

In addition to band overlap in Figure 4e,f, metals (Au) generally exhibit higher carrier concentrations than semimetals (Bi). Therefore, the Au substrate is expected to show a stronger substrate-induced screening effect than the Bi substrate. However, the carrier concentration of MoS_2_ on Bi(111) is much higher than that of MoS_2_ on Au(111) based on the CBM results of Figures 2e and 3e. Therefore, MoS_2_ on the Bi(111) substrate resembles a metallic system (degenerate semiconductor), which represents a stronger carrier-induced screening effect of MoS_2_.^52,53^ Both substrate-induced and carrier-induced screening effects are able to lower the CBM and reduce the band gap (increase the electron affinity).^41,52,53^ The CBM and band gap reduction support the conduction band modulation in MoS_2_, which may also alter the valley structure’s integrity and affect the QPI signal.^41,52,53^ That is to say, substrate-induced and carrier-induced screening effects are likely to impact the QPI signal.

Accordingly, MoS_2_/Au­(111) exhibits a stronger substrate-induced screening effect, while MoS_2_/Bi­(111) shows a stronger carrier-induced screening effect determined by the higher carrier concentration of MoS_2_. To determine which system exhibits the stronger overall screening effect (including substrate-induced and carrier-induced screening effect), we compare the band gap obtained from our experimental measurements in Figures 2e and 3e.^41,52,53^ The band gap of MoS_2_/Bi­(111) is 1.57 eV, which is much smaller than that of MoS_2_/Au­(111) (2.09 eV). Therefore, we conclude that the overall screening effect of MoS_2_/Bi­(111) is stronger, but it still retains and presents the 2 × 2 QPI signal. This indicates that the screening effect is not the primary factor affecting the **Q**-valley electronic structure of MoS_2_/Bi­(111). In contrast, the disappearance of the 2 × 2 QPI signal in MoS_2_/Au­(111) is more likely attributed to severe band overlap, as shown in the CEC of Figure 4e. These observations demonstrate that while the **Q**-valley structure undergoes substantial modification in MoS_2_/Au­(111), it remains largely intact in MoS_2_/Bi­(111), explaining the latter’s superior sheet resistance and contact resistance properties.

Our study identified two key factors enhancing contact performance in the MoS_2_/Bi­(111) system: (1) the relatively narrow MIGS energy distribution, positioned far from the CBM, facilitates gap state saturation below the CBM and promotes ohmic contact formation. (2) The preservation of the **Q**-valley band structure above the CBM reduces intrinsic sheet resistance and enhances transport properties under high carrier concentration conditions.

## Conclusions

In summary, this study investigated electronic structure evolution through d*I*/d*V* characterization of monolayer MoS_2_ in contact with metallic (Au) and semimetallic (Bi) substrates, comparing substrate-contacted and substrate-free regions. This direct experimental approach revealed how interlayer coupling between MoS_2_ and metal/semimetal substrates modulates MIGS.

Quantitative decay length analysis demonstrated substrate-dependent variations in MIGS spatial and energy distributions. Our findings show that MIGS distribution significantly impacts the SBH, with MIGS further from the CBM driving CNL convergence toward the CBM, effectively reducing the SBH. Concurrent analysis of 2 × 2 QPI patterns reveals substrate-dependent **Q**-valley band modifications: metallic substrates cause extensive **Q**-valley band destruction, while semimetallic substrates preserve the intrinsic **Q**-valley structure. This band structure preservation, combined with favorable MIGS characteristics, establishes semimetallic substrates as optimal candidates for enhancing the contact performance in TMD-based electronic devices.

These findings demonstrate that optimal contact engineering can be achieved by selecting semimetals with weak interlayer coupling to the semiconductor conduction band while simultaneously minimizing contact resistance and intrinsic sheet resistance. This structure provides insights into the fundamental mechanisms governing semimetal-2D semiconductor junctions.

## Methods

STM and in situ nc-AFM measurements were performed by low-temperature STM (LT-STM) equipped with a tungsten tip and a QPlus sensor (*f*_0_ = 26000 Hz) in an ultrahigh vacuum environment (below 10^–10^ Torr) and a based temperature of 77K. We used the lock-in technique to measure the curves or images of d*I*/d*V* with bias modulation δ*V* = 5 ∼10 mV, *f* = 700 ∼900 Hz.

## Supplementary Material



## Notes

The VBM/CBM determination of linear regression method, direct comparisons about the in-gap state behavior in both the substrate-contacted and the substrate-free regions of MoS_2_/Au­(111), the detailed d*I*/d*V* spectrum analysis on MoS_2_/Bi­(111), the MIGS density comparison between MoS_2_/Au­(111) and MoS_2_/Bi­(111), and local FT analysis of d*I*/d*V* mapping in pristine and pit regions of MoS_2_/Au­(111) (PDF)
