# Supplementary material for: Direct Visualization of Metal-Induced Gap State Distribution and Valley Band Evolution at Metal Versus Semimetal MoS2 Interfaces
Source: ACS Nano. 2025 May 15;19(20):19408–16. doi: 10.1021/acsnano.5c03676 (PMC12120976; doi:10.1021/acsnano.5c03676)
Supplement: Supplementary file 1 [file nn5c03676_si_001.pdf]

Supporting Information

Direct Visualization of Metal-Induced Gap State Distribution and Valley Band Evolution at Metal Versus Semimetal MoS_2 Interfaces

Yi-Feng Chen[1,2], Hung-Chang Hsu[2], Hao-Yu Chen[1], Liang-Yu Chen[2], Yan-Ruei Lin[1], Ming-Yang Li[3], Iuliana P. Radu[3], Ya-Ping Chiu[*1,2,4,5]

[1] Graduate School of Advanced Technology, National Taiwan University, Taipei 10617, Taiwan

[2] Department of Physics, National Taiwan University, Taipei 10617, Taiwan

[3] Taiwan Semiconductor Manufacturing Company, Hsinchu 30078, Taiwan

[4] Institute of Physics, Academia Sinica, Taipei 115201, Taiwan

[5] Institute of Atomic and Molecular Sciences, Academia Sinica, Taipei 10617, Taiwan

Supporting Information 1

The VBM/CBM of MoS_2 determination

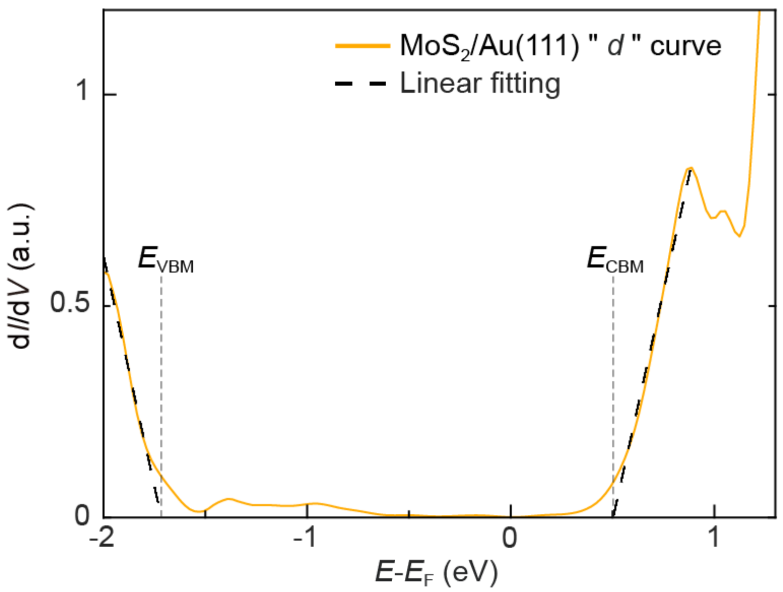

Supporting Figure 1 VBM/CBM determination: The VBM/CBM of MoS_2 are determined from representative dI/dV curves for the substrate-contacted and substrate-free regions. The representative curve for the substrate-free region on MoS_2/Au(111) is shown as the yellow line. Linear fitting is made to dI/dV curve for the specific energy range ΔE, in which the R^2 of the linear fitting will be greater than 0.95. The VBM/CBM is defined as the intersection point of the linear regression curve with dI/dV = 0.

The VBM/CBM of MoS_2 on the substrate-contacted and substrate-free is derived from the representative dI/dV curves "*c*" and "*d*", respectively, through the linear fitting. The curve "*d*" of MoS_2/Au(111), used as an example, is represented by the yellow line in **Supporting Figure 1**. The range for linear regression is confined to the energy intervals on both sides of the E_F, where monotonically increasing dI/dV values are observed. The linear fitting (black dashed line) must achieve an R^2 value greater than 0.95. The VBM/CBM is defined as the intersection point (gray

dashed line) of the linear fitting results and dI/dV = 0.[1]

Supporting Information 2

The consistent in-gap states behavior in the substrate-contacted region of MoS_2/Au(111), and the decreased in-gap states in the substrate-free region

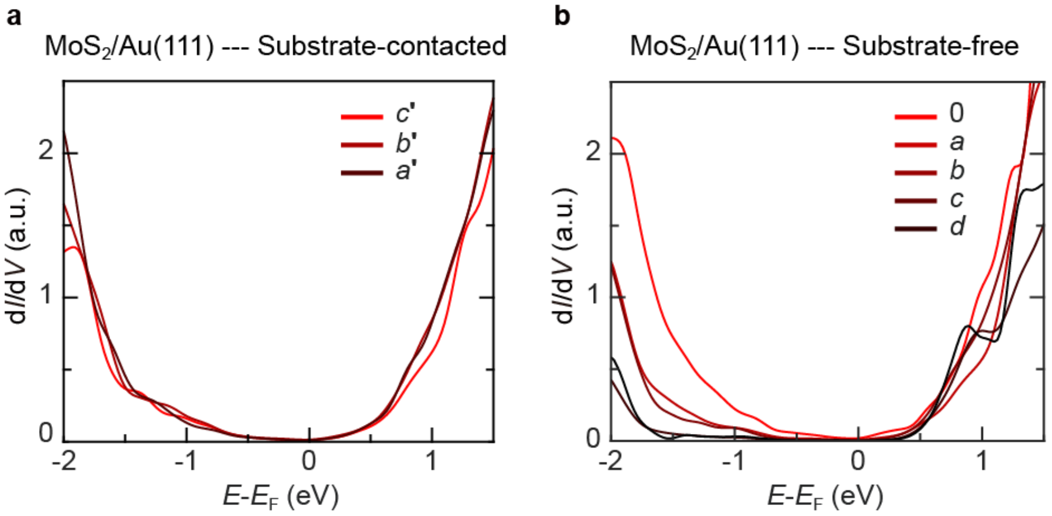

Supporting Figure 2: (a) In the substrate-contacted region, the d*I*/d*V* curves “*a*'” through “*c*'” with a spatial resolution of approximately 0.4 nm are shown and share the same baseline, d*I*/d*V* = 0. (b) From the substrate edge (origin) toward substrate-free region, the d*I*/d*V* curves “0” through “*d*” with a spatial resolution of approximately 0.4 nm are shown and share the same baseline, d*I*/d*V* = 0.

By aligning the “*a*'” to “*c*'” curves sampled at approximately 0.4 nm intervals from the substrate edge to the substrate-contacted region on the same baseline (d*I*/d*V* = 0), as shown in **Supporting Figure 2a**. It is evident that the three curves exhibit similar d*I*/d*V* intensities and variations across different energy levels. On the other hand, the “0” to “*d*” curves sampled at approximately 0.4 nm intervals from the substrate edge to the substrate-free region are shown in **Supporting Figure 2b**. In the substrate-free region of MoS_2, a gradually decreasing in-gap states can be observed within its energy band gap (-1.72 eV to +0.50 eV).

Supporting Information 3

The detailed dI/dV spectrum analysis on MoS_2/Bi(111)

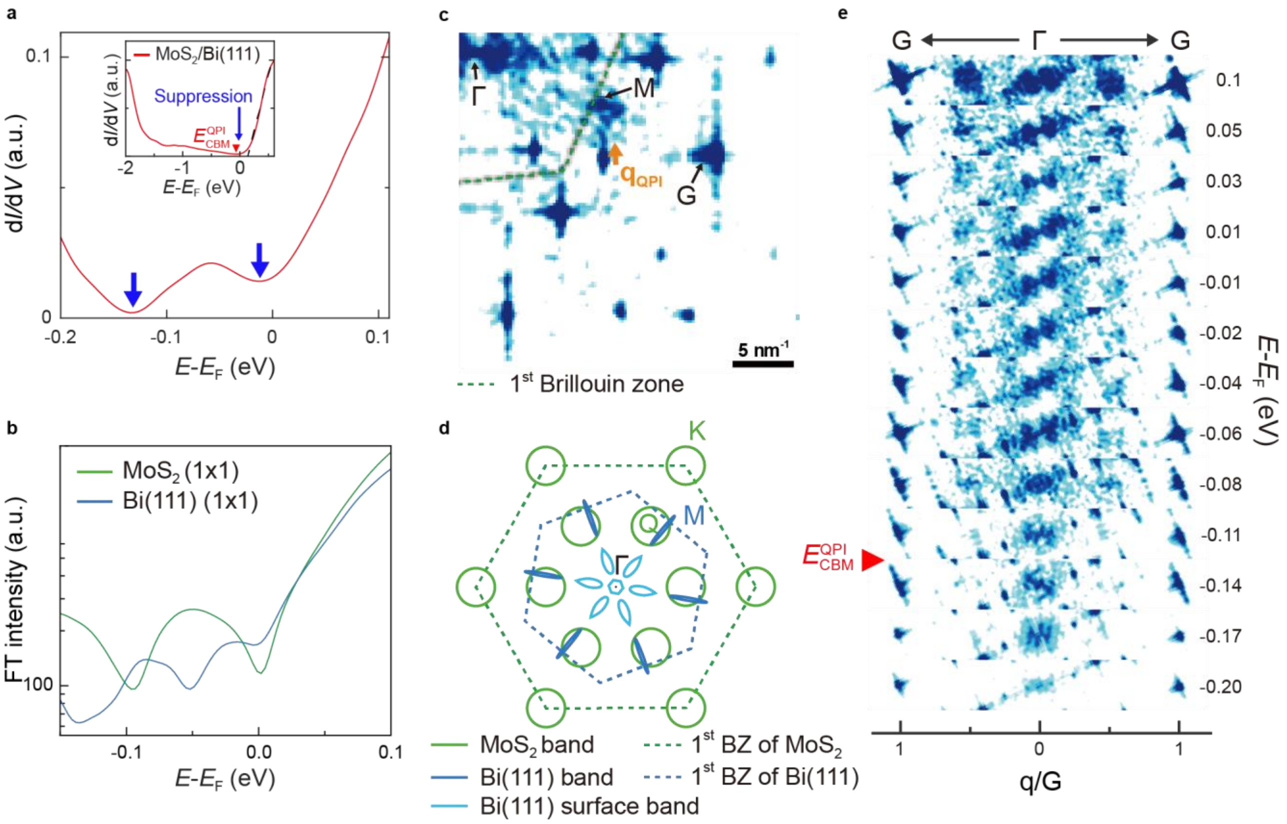

Supporting Figure 3: (a) The detailed dI/dV spectrum with approximate 3.5 meV energy resolution is measured from the substrate-contacted region of MoS_2/Bi(111). The blue arrows show two local minimums near the E_F and -0.13 eV. The inset shows the representative dI/dV spectra in the substrate-contacted region of MoS_2/Bi(111) with LDOS suppression (blue arrow) near the E_F. The CBM of the red curve derived from the linear fitting is higher than that derived from the QPI analysis. (b) The FT intensity is derived from the dI/dV mapping of the substrate-contacted region of MoS_2/Bi(111) under different energy level. (c) The FT image of the dI/dV mapping in the substrate-contacted region of MoS_2/Bi(111) is measured at the V_S = +0.05 V. The green dashed line shows the first BZ of MoS_2, while the orange arrow shows the 2 × 2 QPI pattern. (d) The schematic viewgraph of CEC with the energy level above the E_F shows the MoS_2 and Bi(111) energy bands overlap in reciprocal space, corresponding to the stacking configuration of MoS_2/Bi(111). (e) Energy-dependent FT of MoS_2/Bi(111) near the E_F is collected from the same direction along the reciprocal lattice vector **G**. The 2 × 2 QPI pattern is at **q**/**G** = ±0.5, and the red triangle indicates the CBM, the energy level where the 2 × 2 pattern disappearance.

The detailed dI/dV spectrum in the substrate-contacted region of MoS_2/Bi(111) with 3.5 meV energy resolution is shown in **Supporting Figure 3a**. The local minimum at -0.13 eV and the E_F suggest that the CBM is under the E_F, and the suppressed LDOS near the E_F is due to the high carrier concentration of MoS_2/Bi(111).[2-4] The FT intensity comparison between MoS_2 and Bi(111) in **Supporting Figure 3b** shows that the LDOS of MoS_2 dominates the dI/dV spectrum at the energy level between -0.1 eV and the E_F. It also supports the contribution of the MoS_2 CB at this energy level interval (-0.1 eV to E_F). Near the E_F, the FT image of the dI/dV mapping reveals the additional 2 × 2 QPI pattern occurring near the **M** point of MoS_2's first BZ, as shown in **Supporting Figure 3c**. It results from the intervalley scattering of **Q**-valleys and represents the **Q**-valley preservation under the interlayer coupling between MoS_2 and Bi(111), as shown in **Supporting Figure 3d**.[5] The energy-dependent FT images in **Supporting Figure 3e** indicate that the onset energy level of 2 × 2 QPI signals at **q**/**G** = ±0.5 is at -0.11 eV. Therefore, the **Q**-valley position can be estimated at the -0.11 eV marked by the red triangle. Previous literature indicates that, when the **Q**-valley of MoS_2 is near the energy level of -0.11 eV, the carrier concentration of MoS_2 on Bi(111) will reach up to 10^{13} cm^{-2},[2] consistent with other measurement results.[3] At such a high carrier concentration (10^{13} cm^{-2}), the **K**- and **Q**-valleys of MoS_2 are expected to align, allowing the onset energy level of the 2 × 2 QPI signal (-0.11 eV) to serve as a highly accurate approximation of the actual CBM.[2,3] Moreover, under high carrier concentration (10^{13} cm^{-2}), the inset in **Supporting Figure 3a** shows the representative dI/dV spectra with LDOS suppression near the E_F.[4] The LDOS suppression near the E_F restricts the energy

range available for linear fitting of the monotonically increasing dI/dV curve to energies above the E_F, as marked by the black dashed line. As a result, the CBM from linear regression will overestimate the actual CBM with much more deviation than QPI analysis.

In conclusion, under high carrier concentration in the MoS_2/Bi(111), the QPI analysis originating from the **Q**-valley scattering provides a closer estimation of the actual CBM, whereas the linear regression method tends to produce a larger deviation due to LDOS suppression near the E_F. This leads to a noticeable discrepancy between the QPI analysis and the linear regression method.

Supporting Information 4

The MIGS density comparison between MoS_2/Au(111) and MoS_2/Bi(111)

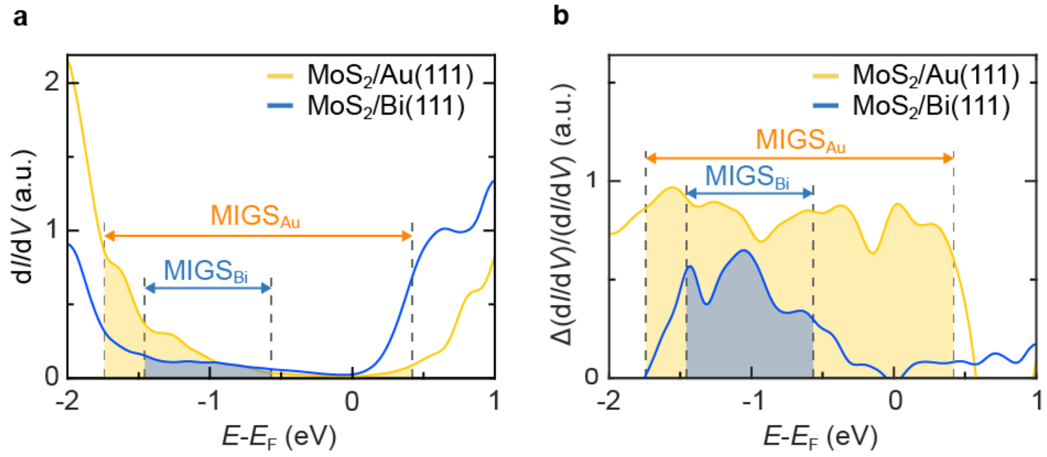

Supporting Figure 4 The MIGS density comparison: (a) The dI/dV derived from the substrate-contacted regions of MoS_2/Au(111) and MoS_2/Bi(111) are shown as the yellow and blue lines, respectively. The MIGS distributions of MoS_2/Au(111) and MoS_2/Bi(111) derived from the decay length analysis are colored by shading yellow and blue, respectively. (b) Δ(dI/dV)/(dI/dV) represents that the dI/dV curve in the substrate-free region (“*d*” curve) subtracted from that in the substrate-contacted region (“*a*” curve) is normalized by the dI/dV curve in the substrate-contacted region. This analysis exhibits the MIGS decay behavior and the relative MIGS density simultaneously.

From the MIGS energy distribution, it is evident that the MIGS in MoS_2/Au(111) (-1.74 eV to +0.41 eV) spreads more widely compared to MoS_2/Bi(111) (-1.46 eV to -0.57 eV). It suggests the higher MIGS spatial density in MoS_2/Au(111). In the corresponding MIGS distribution, the dI/dV intensities of MoS_2/Au(111) are generally higher than that of MoS_2/Bi(111) as shown in **Supporting Figure 4a**. In **Supporting Figure 4b**, the Δ(dI/dV)/(dI/dV) curves roughly represent the proportion of dI/dV intensities contributed by MIGS and the decay length as Δ(dI/dV)/(dI/dV) value is within 0 to 1. Both dI/dV intensities and Δ(dI/dV)/(dI/dV) values in MoS_2/Au(111) are higher than those of

MoS_2/Bi(111). This result supports that MoS_2/Au(111) has a higher MIGS density (per unit energy and per unit area) compared to MoS_2/Bi(111).

Supporting Information 5

Local FT analysis of dI/dV mapping in pristine and pit regions of MoS_2/Au(111)

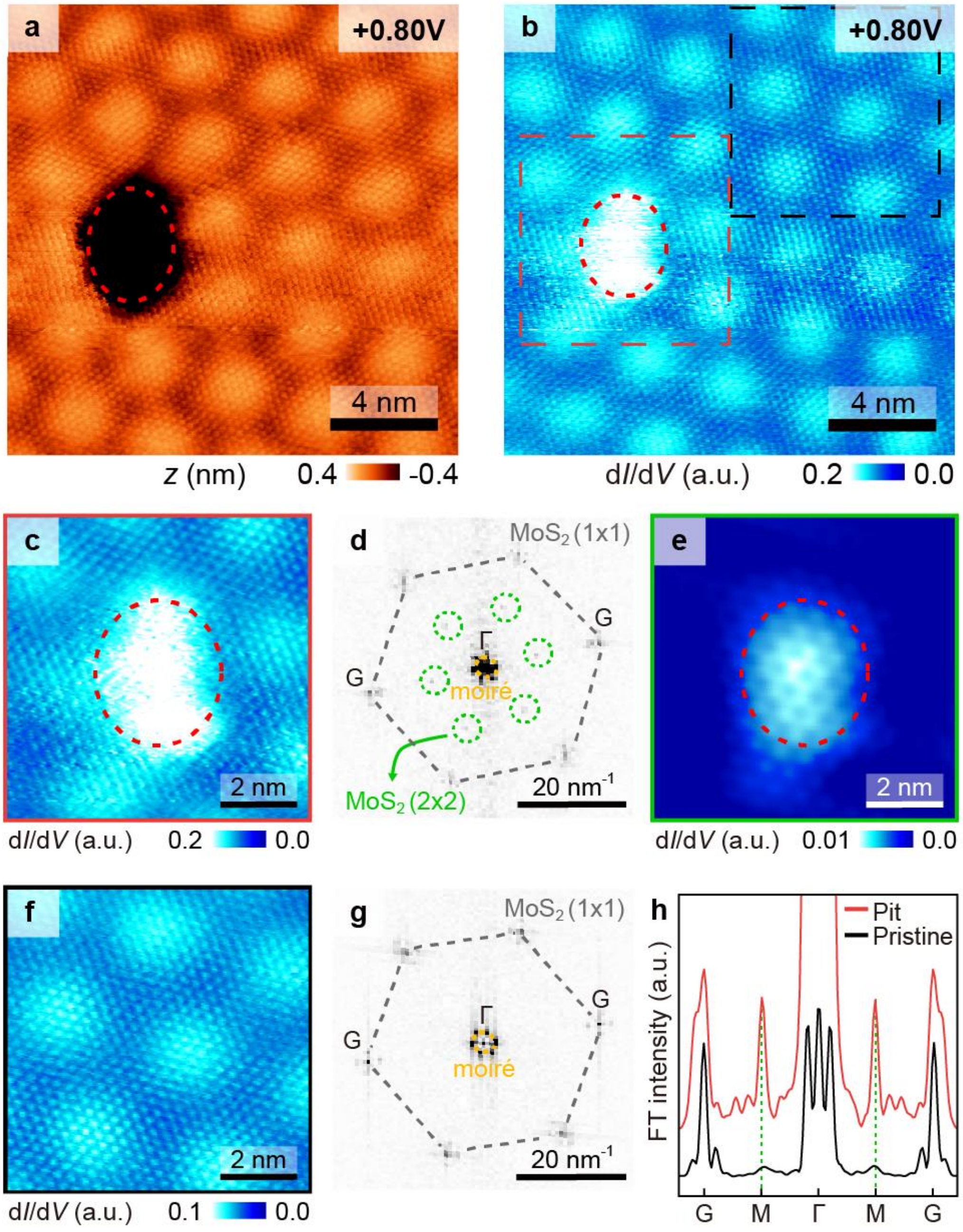

Supporting Figure 5 Local FT analysis of d*I*/d*V* mapping in pristine and pit regions of MoS_2/Au(111): (a) The STM image encompasses both the pristine (substrate-contacted) and pit (substrate-free, marked by the red dashed line) regions with Vs = +0.80 V, Iset = 100 pA. (b) The dI/dV mapping corresponds to (a) with Vs = +0.80 V. The pristine region is marked by the black dashed line, while the pit region is marked by the red dashed line. (c)The local dI/dV mapping corresponds the region marked by the red dashed line in (b). (d) The FT image corresponds to (c). The 2 × 2 QPI signals are marked by the green dashed line. (e) The local dI/dV mapping is derived from (a) with only the remaining FT signal of MoS_2 (2 × 2) and indicates the 2 × 2 QPI signals are concentrated in the pit (marked by the red dashed circle). (f) The

local dI/dV mapping corresponds the region marked by the black dashed line in (b). (g) The FT image corresponds to (f). (h) The FT intensities is collected from (d) and (g) along the direction from the **G** point to **Γ** point with the red and black line, respectively. The 2 × 2 QPI signals only appear in the pit region (red line) at **M** point.

Supporting Figure 5a and **5b** exhibit the STM image and corresponding dI/dV mapping encompassing both the pristine (substrate-contacted) and pit (substrate-free) regions, marked by the black and red dashed lines, respectively. The local enlarged dI/dV images of pit (substrate-free) and pristine (substrate-contacted) regions are shown in **Supporting Figure 5c** and **5f**. The red dashed circle in **Supporting Figure 5b** and **5c** represents the region within the pit (substrate-free region). The additional 2 × 2 signals can be observed in **Supporting Figure 5e** within the red dashed circle after filtering the FT signals of MoS_2 (1 × 1) and moiré pattern in **Supporting Figure 5d**. On the other hand, the **Supporting Figure 5g**, the FT image corresponding to **Supporting Figure 5f**, shows no MoS_2 (2 × 2) signals. The FT profile in **Supporting Figure 5h** also shows the stronger MoS_2 (2 × 2) signals at **M** point in the pit region compared to that in the pristine region.

Reference

(1) Shih, F.-Y.; Wu, Y.-C.; Shih, Y.-S.; Shih, M.-C.; Wu, T.-S.; Ho, P.-H.; Chen, C.-W.; Chen, Y.-F.; Chiu, Y.-P.; Wang, W.-H. Environment-Insensitive and Gate-Controllable Photocurrent Enabled by Bandgap Engineering of MoS_2 Junctions. *Sci. Rep.* **2017**, *7*, 44768.
(2) Ge, Y.; Liu, A. Y. Phonon-Mediated Superconductivity in Electron-Doped Single-Layer MoS_2: A First-Principles Prediction. *Phys. Rev. B* **2013**, *87*, 241408.
(3) Shen, P.-C.; Su, C.; Lin, Y.; Chou, A.-S.; Cheng, C.-C.; Park, J.-H.; Chiu, M.-H.; Lu, A.-Y.; Tang, H.-L.; Tavakoli, M. M.; Pitner, G.; Ji, X.; Cai, Z.; Mao, N.; Wang, J.; Tung, V.; Li, J.; Bokor, J.; Zettl, A.; Wu, C.-I.*; et al.* Ultralow Contact Resistance between Semimetal and Monolayer Semiconductors. *Nature* **2021**, *593*, 211-217.
(4) Song, Y. H.; Jia, Z. Y.; Zhang, D.; Zhu, X. Y.; Shi, Z. Q.; Wang, H.; Zhu, L.; Yuan, Q. Q.; Zhang, H.; Xing, D. Y.; Li, S. C. Observation of Coulomb Gap in the Quantum Spin Hall Candidate Single-Layer 1T'-WTe_2. *Nat. Commun.* **2018**, *9*, 4071.
(5) Chen, H.-Y.; Hsu, H.-C.; Huang, C.-C.; Li, M.-Y.; Li, L.-J.; Chiu, Y.-P. Directly Visualizing Photoinduced Renormalized Momentum-Forbidden Electronic Quantum States in an Atomically Thin Semiconductor. *ACS Nano* **2022**, *16*, 9660-9666.
